# Supplementary material for: Directed-edge-based mining of regular routes for enhanced traffic pattern recognition from travel trajectories
Source: PLoS One. 2025 Dec 18;20(12):e0338954. doi: 10.1371/journal.pone.0338954 (PMC12714192; doi:10.1371/journal.pone.0338954)
Supplement: S1 File — (DOCX) [file pone.0338954.s001.docx]

Supporting Information: Figure

**S1 Fig. 1. Path clustering process diagram**

**S2 Fig. 2. The extracted regular routes from the dataset**


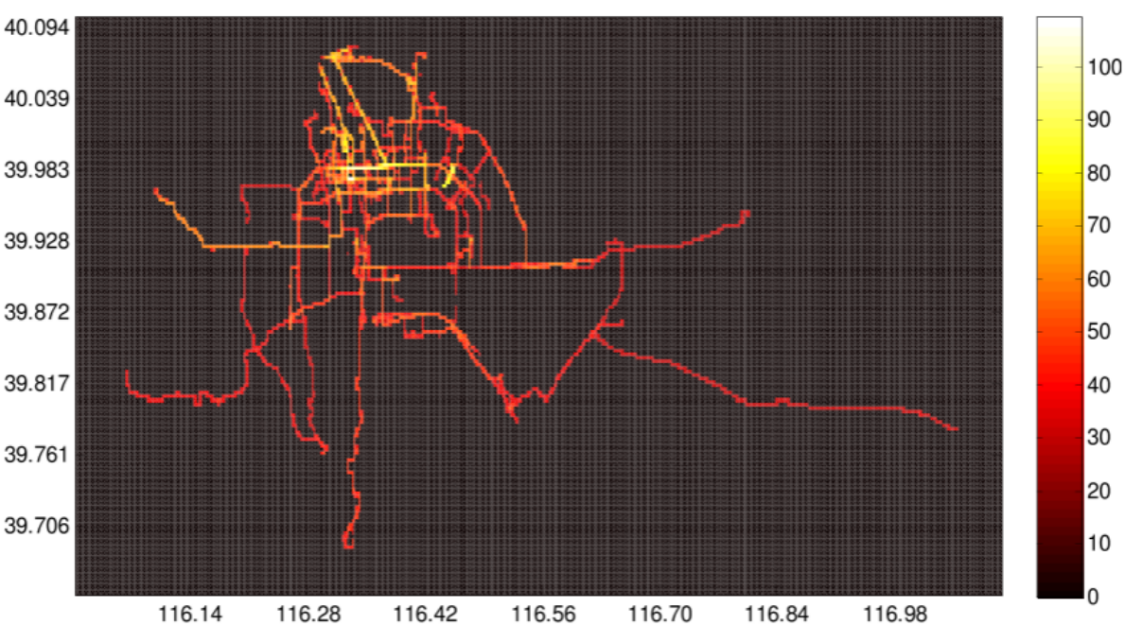


longitude**（^o^）**

latitude**（^o^）**

**S3 Fig. 3. Mapping error variation curve with grid size**

Error distance (m)

Grid size (s)

**S4 Fig. 4. Curve of computing time varying with the number of time sequence grids**


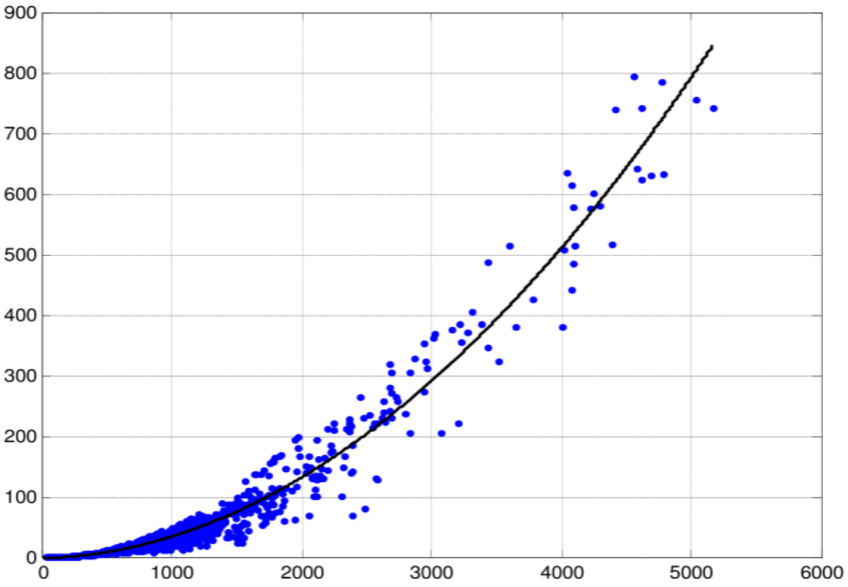


Computing time (ms)

Number of time sequence grids

**S5 Fig. 5. Comparison of MAPE errors among four different algorithms**

#

**S6 Fig. 6. Comparison of Accuracy Ratio of Four Different Algorithms**

**S7 Fig. 7. Comparison of F1 scores for four different algorithms**
