# Supplementary material for: Directed-edge-based mining of regular routes for enhanced traffic pattern recognition from travel trajectories
Source: PLoS One. 2025 Dec 18;20(12):e0338954. doi: 10.1371/journal.pone.0338954 (PMC12714192; doi:10.1371/journal.pone.0338954)
Supplement: S2 File — (DOCX) [file pone.0338954.s002.docx]

Supporting Information: Lab data

**Comparison data of MAPE errors of four different algorithms**

| algorithm |  | | | | | | | | | | |
| --- | --- | --- | --- | --- | --- | --- | --- | --- | --- | --- | --- |
| DeepMove | Test step number | | | | | | | | | | |
|  | 1 | 2 | 3 | 4 | 5 | 6 | 7 | 8 | 9 | 10 | 11 |
|  | MAPE (%) | | | | | | | | | | |
|  | 9.12 | 6.12 | 7.15 | 8.17 | 6.78 | 7.23 | 7.85 | 8.11 | 7.58 | 6.91 | 6.35 |
| TrajCNN | Test step number | | | | | | | | | | |
|  | 1 | 2 | 3 | 4 | 5 | 6 | 7 | 8 | 9 | 10 | 11 |
|  | MAPE (%) | | | | | | | | | | |
|  | 8.32 | 5.63 | 6.42 | 6.39 | 5.13 | 6.62 | 6.78 | 7.02 | 6.89 | 5.23 | 5.11 |
| ST-Transformer | Test step number | | | | | | | | | | |
|  | 1 | 2 | 3 | 4 | 5 | 6 | 7 | 8 | 9 | 10 | 11 |
|  | MAPE (%) | | | | | | | | | | |
|  | 6.35 | 4.78 | 4.31 | 4.35 | 4.21 | 4.87 | 5.02 | 4.93 | 4.87 | 3.93 | 4.35 |
| This article's method | Test step number | | | | | | | | | | |
|  | 1 | 2 | 3 | 4 | 5 | 6 | 7 | 8 | 9 | 10 | 11 |
|  | MAPE (%) | | | | | | | | | | |
|  | 5.13 | 4.15 | 3.16 | 3.41 | 2.15 | 2.74 | 3.01 | 3.11 | 3.02 | 2.11 | 3.24 |

**Comparison data of Accuracy Ratio of Four Different Algorithms**

| algorithm |  | | | | | | | | | | |
| --- | --- | --- | --- | --- | --- | --- | --- | --- | --- | --- | --- |
| DeepMove | Test step number | | | | | | | | | | |
|  | 1 | 2 | 3 | 4 | 5 | 6 | 7 | 8 | 9 | 10 | 11 |
|  | Accuracy ratio | | | | | | | | | | |
|  | 0.48 | 0.57 | 0.64 | 0.71 | 0.66 | 0.71 | 0.73 | 0.58 | 0.63 | 0.68 | 0.69 |
| TrajCNN | Test step number | | | | | | | | | | |
|  | 1 | 2 | 3 | 4 | 5 | 6 | 7 | 8 | 9 | 10 | 11 |
|  | Accuracy ratio | | | | | | | | | | |
|  | 0.51 | 0.64 | 0.69 | 0.74 | 0.72 | 0.75 | 0.79 | 0.62 | 0.68 | 0.72 | 0.74 |
| ST-Transformer | Test step number | | | | | | | | | | |
|  | 1 | 2 | 3 | 4 | 5 | 6 | 7 | 8 | 9 | 10 | 11 |
|  | Accuracy ratio | | | | | | | | | | |
|  | 0.71 | 0.70 | 0.72 | 0.75 | 0.80 | 0.75 | 0.88 | 0.74 | 0.73 | 0.78 | 0.81 |
| This article's method | Test step number | | | | | | | | | | |
|  | 1 | 2 | 3 | 4 | 5 | 6 | 7 | 8 | 9 | 10 | 11 |
|  | Accuracy ratio | | | | | | | | | | |
|  | 0.85 | 0.81 | 0.86 | 0.82 | 0.86 | 0.88 | 0.95 | 0.83 | 0.85 | 0.92 | 0.94 |

**Comparison data of F1 scores of Four Different Algorithms**

| algorithm |  | | | | | | | | | | |
| --- | --- | --- | --- | --- | --- | --- | --- | --- | --- | --- | --- |
| DeepMove | Test step number | | | | | | | | | | |
|  | 1 | 2 | 3 | 4 | 5 | 6 | 7 | 8 | 9 | 10 | 11 |
|  | F1 score | | | | | | | | | | |
|  | 0.58 | 0.61 | 0.65 | 0.73 | 0.69 | 0.74 | 0.76 | 0.68 | 0.73 | 0.74 | 0.68 |
| TrajCNN | Test step number | | | | | | | | | | |
|  | 1 | 2 | 3 | 4 | 5 | 6 | 7 | 8 | 9 | 10 | 11 |
|  | F1 score | | | | | | | | | | |
|  | 0.61 | 0.65 | 0.69 | 0.76 | 0.74 | 0.77 | 0.81 | 0.72 | 0.77 | 0.79 | 0.78 |
| ST-Transformer | Test step number | | | | | | | | | | |
|  | 1 | 2 | 3 | 4 | 5 | 6 | 7 | 8 | 9 | 10 | 11 |
|  | F1 score | | | | | | | | | | |
|  | 0.67 | 0.73 | 0.75 | 0.79 | 0.81 | 0.82 | 0.85 | 0.78 | 0.81 | 0.84 | 0.85 |
| This article's method | Test step number | | | | | | | | | | |
|  | 1 | 2 | 3 | 4 | 5 | 6 | 7 | 8 | 9 | 10 | 11 |
|  | F1 score | | | | | | | | | | |
|  | 0.76 | 0.81 | 0.84 | 0.83 | 0.87 | 0.89 | 0.91 | 0.85 | 0.86 | 0.91 | 0.93 |

**Mapping error variation curve with grid size**

| Grid size (s) | 0 | 5 | 10 | 15 | 20 | 25 | 30 |
| --- | --- | --- | --- | --- | --- | --- | --- |
| Error distance (m) | 0 | 23 | 53 | 77 | 115 | 132 | 162 |
