# Supplementary material for: Directed-edge-based mining of regular routes for enhanced traffic pattern recognition from travel trajectories
Source: PLoS One. 2025 Dec 18;20(12):e0338954. doi: 10.1371/journal.pone.0338954 (PMC12714192; doi:10.1371/journal.pone.0338954)
Supplement: Attachment — Submitted filename: comment_43817.pdf [file pone.0338954.s004.pdf]

1.The research lacks theoretical comparisons and modular contribution analyses with existing advanced methods. It is suggested to quantitatively analyze the contribution degree of each module to the overall performance, and at the same time provide theoretical proofs of the algorithm's time complexity and space complexity.
2.The description of the innovative content in the paper is not clear and definite enough. Meanwhile, the important research achievements in the relevant field in recent years should be reflected in the review. The similarities and differences between them and the method proposed in this paper can be compared to highlight the innovative points.
3.Although the paper briefly mentions the application in dynamic traffic prediction and personalized recommendation, it does not discuss the challenges in actual deployment. It is suggested that the constraints faced in the implementation of the system and its specific value for traffic management be further described.
4.The following studies may be properly cited[1]"An improved artificial potential field method for multi-AGV path planning in ports" Intelligence & Robotics, 2025(5):19-33.[2]Sensing Data Supported Traffic Flow Prediction via Denoising Schemes and ANN: A Comparison.[3] MFMDepth: MetaFormer-based monocular metric depth estimation for distance measurement in ports " Computers & Industrial Engineering, 207(2025), ,111325
