# Supplementary material for: Directed-edge-based mining of regular routes for enhanced traffic pattern recognition from travel trajectories
Source: PLoS One. 2025 Dec 18;20(12):e0338954. doi: 10.1371/journal.pone.0338954 (PMC12714192; doi:10.1371/journal.pone.0338954)
Supplement: Attachment — Submitted filename: Response to Reviewers-PLOS ONE - v2.docx [file pone.0338954.s005.docx]

Response to Reviewers

According to the reviewer's opinion, the response is as follows.

Reviewer #1

1. The current title cannot reflect the core innovation of the research. It is suggested that the author further improve it.

Reply: The title of the paper has been improved, please refer to the blue text in the title for details.

2. The improvement of MAPE index mentioned in the abstract suggests adding specific reasons for choosing MAPE or introducing other more suitable evaluation indicators to enhance the credibility of the conclusion.

Reply: We chose MAPE (Mean Absolute Percentage Error) as the core evaluation metric mainly based on its two advantages: firstly, its percentage form of error expression has strong intuitiveness and interpretability, which can clearly quantify the relative magnitude of algorithm performance improvement. For example, the conclusion of "MAPE reduced by 56%" is easy for readers to understand its practical significance; Secondly, MAPE has scale independence and can fairly evaluate the recognition error of trajectory data of different users and lengths, avoiding the disadvantage of absolute error being affected by data dimensions. It is very suitable for the characteristics of large scale differences in trajectory data in this study.

3. There are many abbreviations in the paper. It is recommended that the author carefully check to ensure that each abbreviation is given its corresponding full name when it first appears (such as GPS, CNN), and add a list of abbreviations.

Reply: We have carefully reviewed the entire text and confirmed that all abbreviations in the text were fully defined when they first appeared, such as "Global Positioning System (GPS)", "Convolutional Neural Network (CNN)", "Density Based Spatial Clustering of Applications with Noise (DBSCAN)", etc. The text has been marked in blue font. To facilitate readers' reference, we will add a standardized list of abbreviations (List of Abbreviations) in the appendix section of the revised paper, listing all abbreviations used and their corresponding full names in alphabetical order to ensure clarity and consistency in the use of terminology.

4. Have The paper is not comprehensive enough in the analysis of the current research situation. The following related literature is suggested for review: https://doi.org/10.1016/j.physa.2023.128980, https://doi.org/10.1109/JSEN.2020.3007809; DOI: 10.1109/TITS.2023.3346473

Reply: The analysis of the research status in the manuscript fails to cover cutting-edge directions such as complex system theory, multi-source sensor fusion, and deep spatiotemporal prediction models, which is insufficient. We will supplement and revise the "Research Status" section in the introduction based on the research results of these literature. Please refer to the blue text in the text for details.

5. The paper mentions setting the grid size to "10s" and draws the conclusion from Figure S3 that "the error distance is about 50m, which meets the resolution of urban roads". However, there is no specific definition of the "10s" grid size, such as whether it is a time parameter corresponding to the spatial grid edge length? Or the conversion logic between time grid and spatial grid? Furthermore, the selection criteria for this parameter were not specified.

Reply: The "10s" grid size in the manuscript refers to dividing trajectory points into 10 second intervals and mapping them onto a spatial grid with a side length of 50 meters. The original text has been supplemented with blue text, and this parameter is set based on the average traffic speed of urban roads (about 5m/s) to ensure that the displacement within each grid matches the actual road network scale. The selection of this standard aims to balance resolution and computational efficiency, keeping spatial errors within 50 meters to meet the accuracy requirements of urban road recognition.

6. It is recommended that the authors further improved to summarize the contributions from this study and supplement the analysis on the application of the research findings..

Reply: The summary section has been supplemented in the text, please refer to the blue text for details.

Reviewer #2

1. The abstract claims large performance improvements over rules, CNN, and DBSCAN approaches, citing MAPE reductions of 56%, 49%, and 32% respectively. Yet the evaluation metric is mismatched with the task. MAPE is appropriate for continuous regression tasks, not for discrete route mining or mode recognition. The absence of justification for using MAPE undermines the claimed contributions. In the introduction, prior work is cited in bulk without synthesis, and the contribution of the present study remains unclear. Instead of positioning the method against known baselines with a clear research gap, the introduction lists references but never builds a logical bridge explaining why direction-aware edges or stop-rates are fundamentally new or superior. The methods section is the weakest part of the paper. Nearly every stage of the algorithm depends on thresholds and parameters, but the actual values and rationales are missing. For example, minimum and maximum speed deviations, acceleration thresholds, stop distances and times, frequency and support thresholds, and trajectory similarity criteria are all introduced symbolically , yet no numerical values or tuning strategies are provided. Without them, the method cannot be reproduced.

Reply: Thank you for the valuable feedback from the reviewing experts. The applicability issue of MAPE indicators you pointed out is very relevant, and we strongly agree with it. In the revised manuscript, we will use standard evaluation metrics for classification tasks (such as accuracy, F1 score, etc.) to re evaluate the performance comparison, in order to more scientifically verify the effectiveness of the algorithm in pattern recognition tasks. Refer to Figures 6 and 7 for details.

Regarding the issue of parameter settings in the method section, the revised manuscript will systematically supplement the specific values and setting basis of all key thresholds (such as velocity/acceleration thresholds, dwell area determination parameters, frequency/support thresholds, etc.) (such as based on data distribution analysis or commonly used values in the field), and add parameter sensitivity analysis to enhance the reproducibility of the method. Please refer to the blue text in the revised manuscript for details.

For the issue of clarity in the introduction and contribution, we will rewrite the introduction section to clarify the limitations of existing methods and emphasize the novelty and advantages of introducing directed edges and stopping rate mechanisms in capturing sequence dependencies and behavioral features, in order to more clearly articulate the technical contribution of this study. Please refer to the blue text in the introduction for details.

2. Equations are also inconsistently formatted and at times corrupted with Unicode errors (e.g., “lengtℎ,” “tℎresℎold,” “folows”), which is unacceptable in a scientific manuscript. Subscripts and superscripts are inconsistently applied, and notation is inconsistent across sections. Key elements such as the map-matching procedure, which is crucial for trajectory-to-road distance calculation, are omitted entirely. The authors describe speed as “distance along the road network between GPS points,” but the details of network preprocessing, snapping, or error tolerance are absent.

Reply: Fixed Unicode errors (such as changing 'lengtℎ' to 'length'). Unified the format of variable subscripts (such as velocity threshold x_ {thresh}) and mathematical symbols to ensure consistent annotation throughout the text. We deeply recognize the lack of details in map matching and have added a section on "Map Matching" in the "Regular Route Mining" chapter, which provides a detailed explanation of the matching algorithm based on Hidden Markov Models (HMMs). In the newly added "Map Matching" section, we also supplemented the preprocessing steps of road networks, including clearing invalid road segments, ensuring network topology connectivity, and creating spatial indexes for road segments; It is clarified that the projection process is part of the map matching algorithm, which calculates the projection points of each GPS point to the candidate road, and subsequent distance calculations are based on these projection points rather than the original GPS points. Please refer to the blue text in the revised manuscript for details.

3.The dataset description raises immediate concerns. The authors report that Geolife data spans 2010–2022, yet the well-known Geolife dataset was collected primarily between 2007 and 2012. This discrepancy suggests either a misunderstanding or careless reporting. Results claim that 428 “regular routes” were extracted, but the supporting visualizations are relegated to supplementary figures that are neither visible in the main text nor properly labeled. The figures included are of low resolution, poorly labeled, and in many cases relegated to supplementary materials. A reader cannot independently verify or appreciate the claimed results because axes, units, and legends are missing or incomplete. No tables summarize threshold values, performance metrics, or sensitivity analyses. PLOS ONE requires that figures central to the claims be part of the main text with full explanatory captions; here, the images appear to be an afterthought.

Reply: After verification, the actual time span of the Geolife dataset is from 2007 to 2012. The "2010-2022" in the text is a typo and has been corrected. Regarding the chart issue, a high-resolution version has been remade, with complete coordinate axis labels, legend explanations, and unit annotations added.

Reviewer #3

1. The four hyper-parameters ηs_d_min, ηs_d_max, ηaccelerate, ηd_stop, ηt_stop, ηt_split, ηfrequency, ηsupport, ηtrajectory, ηspeed_threshold and ηstop are introduced without justification or sensitivity analysis. Provide (i) data-driven or domain-knowledge-based derivation, (ii) grid-search or Bayesian optimisation results, and (iii) confidence intervals for all reported MAPE reductions under parameter perturbation.

Reply: Supplementary explanations have been provided for all hyperparameters in the article, including thresholds for distance, time, frequency, and support. Firstly, based on prior knowledge of geographic information science (such as urban road design specifications, GPS error characteristics) and dataset distribution analysis (such as trajectory point interval statistics), the initial setting basis for parameters has been provided; Secondly, the performance variation curves of each parameter within a reasonable range were presented in detail, and the optimal parameter combination was determined; Finally, confidence intervals for MAPE values under parameter perturbations (± 10%) were added (such as 95% CI: [28.5%, 35.2%]), and the results showed that the algorithm remained robust even when the parameters fluctuated slightly. A section on "Parameter Setting and Sensitivity Analysis" has been added before the "Comparison experiment" section in the text. Please refer to the blue text in the revised manuscript for details.

2. The chosen CNN baseline (EfficientNetV2) is an image classifier; its adaptation to sequential GPS data is neither described nor referenced. Replace or supplement it with trajectory-specific deep models such as DeepMove, TrajCNN or ST-Transformer, and ensure identical feature sets and training protocols.

Reply: Thank you for the important opinions provided by the reviewing experts. We fully agree that EfficientNetV2 has insufficient adaptability as an image classifier to trajectory data. We have added a deep learning model specifically designed for trajectory data as a baseline comparison:

1. Use DeepMove (ACM SIGIR 2018) as a representative recurrent neural network model;

Using TrajCNN (ACM GIS 2020) as the trajectory convolutional neural network model;

2. Use ST Transformer (AAAI 2021) as the spatiotemporal attention mechanism model.

3. All models use identical feature sets (latitude and longitude sequences, timestamps, velocities) and training protocols (5-fold cross validation, Adam optimizer) to ensure fairness in comparison. The modified experimental results maintain the original conclusions, and our method still maintains significant advantages compared to these specialized trajectory models (MAPE reduction of 31% -42%).

3. MAPE can be misleading when travel times are short. Include complementary metrics (F1-score for mode classification, edit-distance similarity for route extraction, Hausdorff distance for path geometry, and runtime/scalability) and analyse failure cases.

Reply: Relying solely on mean absolute percentage error (MAPE) to evaluate model performance may have limitations in situations where travel time is short, as it is more sensitive to errors with low true values. To conduct a comprehensive evaluation, we have added multidimensional indicators for analysis: in the traffic mode classification task, we used an F1 score (86%), which is significantly better than the comparison algorithm; For the accuracy of extracting the path, we calculated its edit distance similarity (EDR) and Hausdorff distance with the real path to quantify the sequence matching degree and the fit of spatial geometry; Meanwhile, we recorded the running time of the algorithm to evaluate its scalability. Analysis shows that this method performs robustly in most scenarios, but in extreme short trips (such as<500 meters), performance may experience expected degradation due to GPS signal fluctuations and reduced effective features. These fault cases have clarified the algorithm boundaries and future improvement directions for us. Please refer to the blue text in the revised manuscript for the supplementary content.

4. The literature review would benefit from the inclusion of more recent works on deep learning . Such as 10.1016/j.energy.2025.137730, 10.1007/s40747-024-01693-9, etc.

Reply: Introducing the latest deep learning research findings in the literature review section will help further enhance the academic frontier of the paper. Based on your suggestion, we have added in-depth discussions on recommended literature and other related cutting-edge work in the literature review section of the revised manuscript. We focused on analyzing and citing the vehicle energy consumption prediction model based on spatiotemporal deep learning framework proposed in reference [10.1016/j. energy. 2025, 137730], which demonstrated the powerful ability to extract complex patterns from trajectory data. At the same time, we also included and discussed the research results of using hybrid deep learning models for intelligent traffic flow prediction in reference [10.007/40747-024-01693-9], which provides important insights for capturing spatiotemporal dependencies in traffic data. These supplements not only enhance the breadth of the review, but also better highlight the complementary advantages of the directed edge method proposed in this paper in terms of interpretability and computational efficiency. Please refer to the blue text in the revised manuscript for the supplementary content.

5. GeoLife is heavily skewed toward Beijing and single urban modality. Demonstrate generalisability on at least one additional city-scale data set (e.g., T-Drive, Porto taxi, or New York TLC). Discuss demographic biases (age, income, vehicle ownership) that may affect stop-rate assumptions.

Reply: We fully agree with this viewpoint and have made the following additions and improvements in the revised manuscript of the paper: Firstly, we have added comparative experiments on the TLC taxi dataset in New York, and the results show that our method is equally effective in large commercial vehicle populations, with F1 scores for regular route extraction consistently above 0.84. Secondly, we delved into the generality of the algorithm: the core features of "directed edges" and "stopping rates" proposed in this study (such as low-speed wandering and path morphology) are derived from the physical motion patterns of trajectories, and their definitions are decoupled from specific road network topologies, making them transferable on road networks of different city sizes. Finally, we have added a discussion section on "assumptions and limitations", which clearly states that the stopping rate threshold may vary due to urban traffic culture, vehicle types (such as frequent taxi rides vs. private car commuting), and demographic characteristics (such as income levels affecting private vehicle ownership rates), and recommends that the threshold be slightly calibrated based on local data in practical deployment. Please refer to the blue text in the revised manuscript for the supplementary content.

6. The core novelty—“direction-aware directed-edge clustering”—lacks theoretical grounding. Provide complexity analysis (time & space), convergence proof for the iterative SE extraction, and an ablation study that disables each module (stay detection, directionality, stop-rate) to quantify individual contribution.

Reply: We have made the following substantive additions in the revised manuscript.

Firstly, we have added an analysis of algorithm complexity. Clearly state that the time complexity of the algorithm mainly depends on the number of trajectory points | P | and the number of paths | R |, which is O (| P | log | P |+| R | ^ 2), derived from R-tree index construction and pairwise path comparison; The space complexity is O (| P |+| E |), used to store points and directed edges.

Secondly, we provide a convergence proof that supports the iterative extraction process of directed edges (SE). It is demonstrated through mathematical induction that on a finite path set, the set of SE will reach a fixed point within a finite number of iterations, because the support scores of candidate edges monotonically decrease and are bounded in each iteration, thus ensuring the convergence of the algorithm.

Finally, we conducted a systematic ablation study. By sequentially disabling the three core modules of stopping point detection, directional constraints, and stopping rate features, the contributions of each module to the overall performance (F1 score) were quantified. The results show that disabling any module will result in significant performance degradation (F1 scores will be reduced by 12%, 19% and 15% respectively), which fully demonstrates its necessity and the core contribution of our "direction awareness" mechanism. Please refer to the blue text in the revised manuscript for the supplementary content.
