# Supplementary material for: Directed-edge-based mining of regular routes for enhanced traffic pattern recognition from travel trajectories
Source: PLoS One. 2025 Dec 18;20(12):e0338954. doi: 10.1371/journal.pone.0338954 (PMC12714192; doi:10.1371/journal.pone.0338954)
Supplement: Attachment — Submitted filename: Response to Reviewers-PLOS ONE - v4.docx [file pone.0338954.s006.docx]

Response to Reviewers

According to the reviewer's opinion, the response is as follows.

Reviewer #1

1.The research lacks theoretical comparisons and modular contribution analyses with existing advanced methods. It is suggested to quantitatively analyze the contribution degree of each module to the overall performance, and at the same time provide theoretical proofs of the algorithm's time complexity and space complexity.

Reply: In response to the expert review, we have significantly strengthened our paper by conducting a comprehensive ablation study which quantitatively demonstrates the critical contributions of each module: removing stay-point detection, directionality, and the stop-rate feature caused F1-score drops of 12%, 19%, and 15%, respectively. We further provide rigorous theoretical analyses, establishing the algorithm's time complexity as O(|P|log|P| + |R|²) and proving its convergence, while explicit comparisons with advanced baselines and cross-dataset validation on NYC TLC data (F1=0.82) conclusively validate our method's superiority, theoretical soundness, and strong generalization capability. Please refer to the blue text in the manuscript for details

2. The description of the innovative content in the paper is not clear and definite enough. Meanwhile, the important research achievements in the relevant field in recent years should be reflected in the review. The similarities and differences between them and the method proposed in this paper can be compared to highlight the innovative points.

Reply: To better highlight our innovative contributions, we will significantly revise the introduction and related work sections. Specifically, we will explicitly state that our core innovation is a directed-edge-based representation that inherently captures the topological sequence and directionality of movement, directly addressing the limitation of point/grid-based methods in modeling continuous path logic. Furthermore, we will expand the literature review to include and discuss recent key achievements in traffic pattern analysis, such as the complex network model for traffic dynamics, the multi-sensor fusion framework, the adaptive spatiotemporal graph convolutional network, the spatiotemporal deep learning model for energy prediction , and the hybrid deep learning model for traffic flow prediction. A dedicated theoretical comparison will be added to clarify the fundamental differences: while these advanced methods excel in network dynamics modeling, state perception, or flow prediction at a system level, our work is novel in its fine-grained, trajectory-level focus on extracting individual regular routes by mining supportive directed edges and introducing the stop-rate feature, a physically-grounded metric for distinguishing transport modes. This contrast will more definitively position the uniqueness of our methodology within the broader research landscape. Please refer to the blue text in the manuscript for details.

3. Although the paper briefly mentions the application in dynamic traffic prediction and personalized recommendation, it does not discuss the challenges in actual deployment. It is suggested that the constraints faced in the implementation of the system and its specific value for traffic management be further described.

Reply: We thank the reviewer for this insightful comment. To address this, we will expand the "Conclusions" section to explicitly discuss the practical challenges and implementation value. The primary deployment constraints include the computational overhead for real-time route extraction on large-scale urban data, which we mitigate through R-tree indexing and complexity of O(|P|log|P| + |R|²), and the need for calibration of key thresholds (e.g., stop-rate) for different urban contexts, which we acknowledge as a limitation requiring local tuning. Regarding its specific value for traffic management, we will elaborate that our method provides fine-grained, individual-level insights into recurrent travel patterns and mode choices, enabling more accurate dynamic traffic prediction at the corridor level by understanding the composition of traffic flows, and facilitating highly personalized navigation and smart travel recommendations that account for a user's habitual routes and preferred transport modes, thereby improving urban mobility efficiency beyond aggregate-level models. Please refer to the blue text in the conclusion section of the manuscript for specific details

4. The following studies may be properly cited[1]"An improved artificial potential field method for multi-AGV path planning in ports" Intelligence & Robotics, 2025(5):19-33. [2]Sensing Data Supported Traffic Flow Prediction via Denoising Schemes and ANN: A Comparison. [3] MFMDepth: MetaFormer-based monocular metric depth estimation for distance measurement in ports " Computers & Industrial Engineering, 207(2025), ,111325

Reply: We sincerely thank the reviewer for suggesting these highly relevant and recent studies. We will incorporate all three recommended citations into our revised manuscript to better position our work within the current research landscape. Specifically, we will cite the improved artificial potential field method for multi-AGV path planning [Suggested Ref 1] in our introduction or related work to contrast its focus on centralized, collision-free routing in controlled environments like ports with our method's core objective of discovering individual, emergent regular routes from noisy GPS data in open urban networks. We will discuss the traffic flow prediction study using denoising and ANN [Suggested Ref 2] to highlight a complementary macro-level approach to traffic modeling that contrasts with our fine-grained, trajectory-level pattern recognition. Finally, we will reference the MFMDepth paper on monocular depth estimation for port distance measurement [Suggested Ref 3] to acknowledge parallel advancements in sensor-based precision localization, thereby underscoring the distinct contribution of our method, which operates effectively on standard GPS data without requiring specialized sensory input for its core route mining functionality. Please refer to the blue text in the introduction and conclusion section of the manuscript for specific details.

Reviewer #3

1. The authors have adequately addressed my previous comments and the manuscript is now suitable for publication. However, please note a discrepancy in Reference 22: the entry in the reference list is authored by **Chen**, while the in-text citation refers to **Huang** — please verify and correct either the in-text citation or the reference list entry so that they match and conform to the journal’s reference style. After this minor correction I recommend acceptance for publication.

Reply: We have made appropriate revisions to the manuscript based on the opinions of the reviewing experts, as indicated in the blue text in the introduction section.
