# Supplementary material for: Directed-edge-based mining of regular routes for enhanced traffic pattern recognition from travel trajectories
Source: PLoS One. 2025 Dec 18;20(12):e0338954. doi: 10.1371/journal.pone.0338954 (PMC12714192; doi:10.1371/journal.pone.0338954)
Supplement: Attachment — Submitted filename: Response to Reviewers-PLOS ONE - v5.docx [file pone.0338954.s007.docx]

Response to Reviewers

According to the reviewer's opinion, the response is as follows.

Reviewer #3

1. The mathematical symbols in the manuscript have not been properly formatted using the appropriate tools

Reply: The mathematical symbols have been modified using appropriate tools, as indicated by the blue symbols in the manuscript.

2. Upon reviewing the manuscript, I found that reference [22] is incorrect. Both the title and authors are inaccurate. The correct article is titled “Predictive modeling and multi-objective optimization of magnetic core loss with activation function flexibly selected Kolmogorov-Arnold networks”, and its DOI is https://doi.org/10.1016/j.energy.2025.137730. Please use Google Scholar to locate the full citation in the proper format. Additionally, please ensure that the following paper is correctly cited: https://doi.org/10.1016/j.knosys.2025.114514. This paper is valuable for describing machine learning concepts.

Reply: Reference [22] has been revised and new references have been cited. Please refer to the blue text in the references for details.
